# Genetic Expression Screening of Arsenic Trioxide-Induced Cytotoxicity in KG-1a Cells Based on Bioinformatics Technology

**DOI:** 10.3389/fgene.2021.654826

**Published:** 2021-08-03

**Authors:** Yahui Li, Yingjie Feng, Xiaohui Si, Chenjin Zhao, Fanping Wang, Xinqing Niu

**Affiliations:** ^1^School of Laboratory Medicine, Xinxiang Medical University, Xinxiang, China; ^2^The Third Affiliated Hospital of Xinxiang Medical University, Xinxiang, China

**Keywords:** arsenic trioxide, acute myeloid leukemia, proliferation, cell cycle, apoptosis, transcriptome

## Abstract

Acute myeloid leukemia (AML) is a malignant tumor of the hematopoietic system, and leukemia stem cells are responsible for AML chemoresistance and relapse. KG-1a cell is considered a leukemia stem cell-enriched cell line, which is resistant to chemotherapy. Arsenic trioxide (ATO) is effective against acute promyelocytic leukemia as a first-line treatment agent, even as remission induction of relapsed cases. ATO has a cytotoxic effect on KG-1a cells, but the mechanism remains unclear. Our results demonstrated that ATO can inhibit cell proliferation, induce apoptosis, and arrest KG-1a cells in the G2/M phase. Using transcriptome analysis, we investigated the candidate target genes regulated by ATO in KG-1a cells. The expression profile analysis showed that the ATO had significantly changed gene expression related to proliferation, apoptosis, and cell cycle. Moreover, MYC, PCNA, and MCM7 were identified as crucial hub genes through protein–protein interaction network analysis; meanwhile, the expressions of them in both RNA and protein levels are down-regulated as confirmed by quantitative polymerase chain reaction and Western blot. Thus, our study suggests that ATO not only inhibits the expression of MYC, PCNA, and MCM7 but also leads to cell cycle arrest and apoptosis in KG-1a cells. Overall, this study provided reliable clues for improving the ATO efficacy in AML.

## Introduction

Acute myeloid leukemia (AML) is a type of malignant clonal disease originating from hematopoietic stem cells, which are highly heterogeneous (Dohner et al., 2015). With the recent development of various therapies, complete remission and overall survival of AML has greatly improved. However, the 5-year survival rate of AML Non-Acute promyelocytic leukemia is still less than 30%, and most patients will eventually relapse (Pullarkat and Aldoss, 2015). The leukemic stem cells (LSCs), possessing extensive self-renewal, differentiation, and infinite proliferation capacity, are responsible for AML refractory and relapse after treatment (Bonnet and Dick, 1997; Thomas and Majeti, 2017). KG-1a cell line, derived from a male AML patient, has the characteristics of leukemia stem cells, such as self-renewal, differentiation, and the phenotype of CD34^+^CD38^–^. Moreover, KG-1a cells are resistant to chemotherapy and natural killer cell-mediated cytotoxicity. Altogether, KG-1a cells are considered as an LSC-enriched cell line (She et al., 2012; Peng et al., 2018). The study of Chen showed that KG-1a cells were resistant to both doxorubicin and arabinoside and a combination of chemotherapy (Chen et al., 2018).

Arsenic trioxide (ATO) combined with all-*trans* retinoic acid has been used clinically for the treatment of PML-RARα-positive acute promyelocytic leukemia, achieving the current remarkable cure rates (Jeanne et al., 2010). Numerous studies have shown that ATO is a multitargeted chemotherapeutic agent that is also effective against many other hematologic malignancies and solid tumors (Kozono et al., 2018; Wang et al., 2019). Our previous study found that ATO has a certain inhibitory effect on the proliferation of KG-1a cells (She et al., 2012), but the mechanism remains unclear. In recent years, transcriptome sequencing technology has been widely used in life science (Wang et al., 2009). Using transcriptome sequencing technology, a previous study about AML revealed that TRPM4 being the only gene encoding a surface protein up-regulated in four AML cell lines after induction by azacitidine treatment (Leung et al., 2019). So far, there is no report about the gene expression profile of KG-1a cells induced by ATO treatment using transcriptome sequencing.

To further explore the role of ATO in AML and the key target genes regulated by ATO, KG-1a cells were divided into ATO-treated group and control group and transcriptome sequencing was performed. This study showed that ATO induced significant antiproliferative effects and obvious apoptosis in KG-1a cells through inhibiting the expression of MYC, PCNA, MCM7, and BCL2L1.

## Materials and Methods

### Cell Proliferation, Apoptosis, and Cell Cycle Analyses

The KG-1a, HL-60, KG-1, and THP-1 cell line was obtained from cryopreserved cell line in our laboratory, which was cultured at 37°C in a 5% CO_2_ atmosphere in RPMI-1640 (Gibco, United States) supplemented with 10% fetal bovine serum (Gibco, United States). The cell culture medium was changed every 1–2 days. All cells used in experiments were in the logarithmic growth phase. All experiments were repeated three times.

According to the instructions of the manufacturer of Cell Counting Kit-8 (CCK8) (Dojindo, Japan), 8 × 10^3^ cells per well were inoculated into a 96-well plate, and the total volume of each well was 100 μL. The KG-1a cells were divided into blank group (no cells or drugs), control group (KG-1a cells and no drugs), and ATO (1, 1.5, 2, 2.5, 3, 3.5, 4, 4.5, 5 μmol/L) groups with different treatments. Each group was provided with three multiple wells, and phosphate-buffered saline (PBS) was added into the edge wells. After 24 h, 10 μL CCK-8 solution was added to each well and cultured in 37°C, 5% CO_2_, and saturated humidity incubator for 1 h. Then, the OD value at 450 nm was detected by the Enzyme Reader, and the average value of 3 wells was taken as the final result. The experiment was repeated three times.

For cell apoptosis analysis, the KG-1a cells in good growth condition were selected and inoculated into the six-well-plate with 1 × 10^6^ cells per well. The KG-1a cells that were divided into blank group, fluorescein isothiocyanate (FITC)–annexin V staining group, propidium iodide (PI) staining group, and experimental group (0, 0.5, 1, 1.5, 2, 2.5, and 3 μmol/L ATO) underwent a series of standardized processes based on the instructions of the manufacturer (FITC–Annexin V Apoptosis Detection Kit, BD Biosciences, United States) to assess the level of apoptosis as follows. After 24 h of conventional culture, KG-1a cells were washed with PBS once. Each tube was gently suspended with 1 mL of binding solution, 100 μL solution was transferred to the EP tube, and 5 μL FITC–annexin V dye solution was added in room temperature protected from light staining for 15 min, during which each tube was slightly vortexed several times; 5 μL PI dye solution was added 5 min before the operation. The cells were filtered and detected on the flow cytometry (BD FACS Calibur, United States). The experiment was repeated three times. The data processing was performed on the FlowJo 7.6.1 software.

For cell cycle analysis, the KG-1a cells in good growth condition were selected and inoculated into the six-well plate with 1 × 10^6^ cells per well. The KG-1a cells that were divided into blank group and experimental group (0, 0.5, 1, 1.5, 2, 2.5, and 3 μmol/L ATO) underwent a series of standardized processes according to PI-staining routine as follows. After 24 h of conventional culture, KG-1a cells were washed with PBS, fixed with precooled 70% alcohol overnight, added 5 μL PI dye solution, and incubated in 37°C water for 30 min; after that, the stained KG-1a cells were detected on the flow cytometry (BD FACS Calibur, United States), and cell cycle phases were analyzed using the ModFit LT 32 software. The experiment was repeated three times.

### RNA Sequencing and Analysis

For RNA sequencing (RNA-seq), the KG-1a cells in good growth condition were selected and inoculated into the six-well-plate with 1 × 10^6^ cells per well, and the KG-1a cells were divided into the control group (no drugs) and the ATO group (2 μmol/L ATO for 24 h); each group had three duplicates. We collected KG-1a cells into six tubes, which were sent to Beijing Novogene Technology Company for transcriptome analysis. In the company, total RNA was extracted by Trizol method. After purification, repairing, and polymerase chain reaction (PCR) amplification, sequencing library construction was completed. After passing the quality inspection, sequencing was carried out on the computer. After obtaining the original data, Casava software was used to detect and filter, and high-quality clean reads were retained. After comparing clean reads with reference sequences, FPKM values were calculated for quantitative analysis. The differentially expressed genes (DEGs) of the ATO group and the control group were analyzed using the DESeq2 R package (1.16.1). The screening threshold is corrected, *p* < 0.05, and | log2(fold change)| ≥ 0.

### Functional Enrichment Analysis and Network Analysis

Functional enrichment of DEGs included enrichment analyses of Gene Ontology (GO) and Kyoto Encyclopedia of Genes and Genomes (KEGG). The GO terms and KEGG terms with corrected *p* < 0.05 were considered as significantly enriched.

Protein--protein interaction (PPI) analysis of DEGs was based on the STRING database,^1^ which contains known and predicted PPIs. And we focused only on the terms related to apoptosis and cell cycle and picked up the DEGs participating in these terms; after that, we used the STRING database to get the PPI network. The PPI network analyses were structured and optimized in the Cytoscape software; also, we used this software to analyze the topological structure of the gene interaction network, through calculating degree, closeness, and betweenness in order to get the genes with the highest degree of connectivity, which means the genes with the most extensive interaction with other genes also mean hub gene. This is shown in Table 1. Moreover, we showed some star molecule and important hub genes in apoptosis and cell cycle regulation in Figure 2B.

**TABLE 1:** Top 10 key hub genes.

**Name**	**log2 Fold change**	**Adjusted *p***	**Degree**	**Closeness**	**Betweenness**
MCM7	−1.94	1.02E−85	36	82.78	538.00
MYC	−1.80	8.56E−69	30	83.00	1,861.57
PCNA	−0.67	2.43E−08	47	89.53	1,817.15
CDC45	−0.63	1.03E−11	33	79.62	354.10
MCM3	−0.58	4.52E−09	36	82.23	584.87
MCM4	−0.49	9.45E−06	39	84.07	645.21
POLE2	−0.48	5.30E−03	32	81.20	428.85
POLE	−0.45	4.15E−06	33	81.03	420.52
RFC3	−0.43	8.25E−03	37	82.37	317.21
FEN1	−0.37	6.91E−04	35	81.07	663.42

### Validation of RNA-Seq Using Reverse Transcription–qPCR

For the real-time fluorescence quantitative PCR [reverse transcription (RT)–qPCR], the KG-1a cells, HL-60 cells, KG-1 cells, and THP-1 cells in good growth condition were selected and inoculated into the six-well-plate with 1 × 10^6^ cells per well. The total RNA was extracted using a traditional method of TRIzol (Cwbio, China) according to the instructions of the manufacturer. Validation of RNA-seq on 10 genes was performed using the RT-qPCR. RT and qPCR reactions were performed using the PrimeScript RT Master Mix (Takara, Japan) and the TB Green Premix Ex Taq II (Takara, Japan). The qPCR reaction was performed using the PikoReal (Thermo Fisher Scientific, United States), and qPCR data were analyzed by the ΔΔCt method. Primers used for RT-qPCR are listed in Supplementary Table 1.

### Validation of RNA-Seq Using Western Blot

The KG-1a cells, HL-60 cells, KG-1 cells, and THP-1 cells were harvested and lysed with RIPA Lysis Buffer (Beyotime, China) supplemented with protease inhibitors (Cwbio, China). After that, the protein concentration was determined and diluted to the same concentration by BCA Detection Kit (Beyotime, China). The equal volume of protein was added in 10% sodium dodecyl sulfate–polyacrylamide gel electrophoresis (Beyotime, China), separated completely, and then transferred onto polyvinylidene fluoride membranes (Millipore, United States). After incubation with 5% skimmed milk to block unspecific binding sites, target proteins were examined using antibodies listed in Supplementary Table 2. Then, the polyvinylidene fluoride membranes were incubated with horseradish peroxidase (HRP)–conjugated secondary antibody (Cwbio, China) and visualized using Amersham Imager 600 (Cytiva, United States) with the HRP substrate (Millipore, United States).

### Statistical Analysis

All experiment data were obtained from at least three independent experiments and presented as the mean ± SD. Statistical analysis was calculated using Kruskal–Wallis *H*-test with Steel–Dwass multiple-contrast test using the GraphPad Prism software. *p* < 0.05 was considered as statistically significant.

## Results

### ATO Inhibits Cell Proliferation, Induces Apoptosis, and Blocks Cell Cycle in KG-1a Cells

**FIGURE 1:**
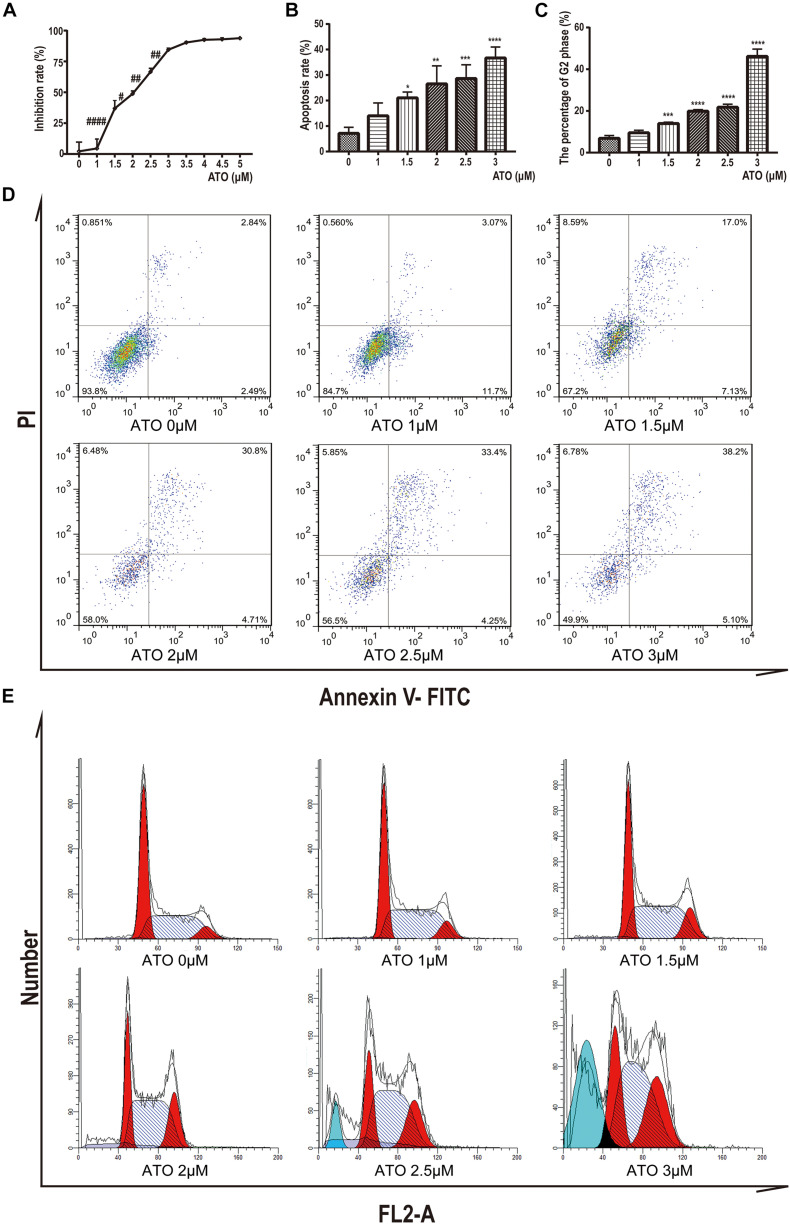
Effects of ATO treatment on proliferation, apoptosis, and cell cycle in KG1a cells. **(A)** Effects of ATO treatment on proliferation in KG1a cells. Cell proliferation was determined using Cell Counting Kit-8 assays, and the cell inhibition rate is shown. Data are shown as means ± SD of three independent experiments. ^#^*p* < 0.05, ^##^*p* < 0.01, ^####^*p* < 0.0001 ATO versus ATO. **(B**,**D)** Effects of ATO treatment on apoptosis in KG1a cells. KG1a cells treated with ATO were analyzed by flow cytometry following annexin V/PI staining; representative scatterplots of KG1a cells treated with ATO are shown **(B)**. The data are expressed as the percentage of cells in annexin V^+^/PI^–^ and annexin V^+^/PI^+^ **(D)**. Data are shown as means ± SD of three independent experiments. *p < 0.05, **p < 0.01, ***p < 0.001, ****p < 0.0001, ATO versus control. **(C**,**E)** Effects of ATO treatment on cell cycle progression in KG1a cells. Flow cytometric analysis of the cell cycle following PI staining; representative histograms of KG1a cells treated with ATO are shown **(C)**. The data are expressed as the percentage of cells in the G2 phase of the cell cycle **(E)**. Data are shown as means ± SD of three independent experiments. ***p < 0.001, ****p < 0.0001, ATO versus control.

To determine the potential effects of ATO, KG-1a cells were treated with different concentrations of ATO ranging from 1 to 5 μM. After 24 h, we performed the cell proliferation assays by the CCK-8 method. We found that ATO has a dosage-dependent inhibitory effect on the proliferation of KG-1a cells (Figure 1A).

To further characterize the mechanisms of growth inhibition by ATO, we analyzed apoptosis after ATO treatment of KG-1a cells. We labeled KG-1a cells with annexin V and PI and observed the proportion of apoptotic cells by flow cytometry. The results showed that apoptotic rate increased significantly in the ATO treatment group compared to the control group (Figures 1B,D).

Moreover, we labeled KG-1a cells with PI to demonstrate the effect of ATO on the cell cycle proportion. Figures 1C,E indicate that KG-1a cells arrested in the G2/M phase when treated with ATO. When ATO concentration reached 2.5 or 3 μmol/L (Figure 1E), a sub-G1 peak represented the apoptotic cells that appeared, which confirmed apoptosis results. In summary, these data indicate that ATO has a cytotoxic effect in KG-1a cells by inducing apoptosis and cell cycle arrest.

### Gene Expression Profiling of ATO-Treated KG-1a Cells

**FIGURE 2:**
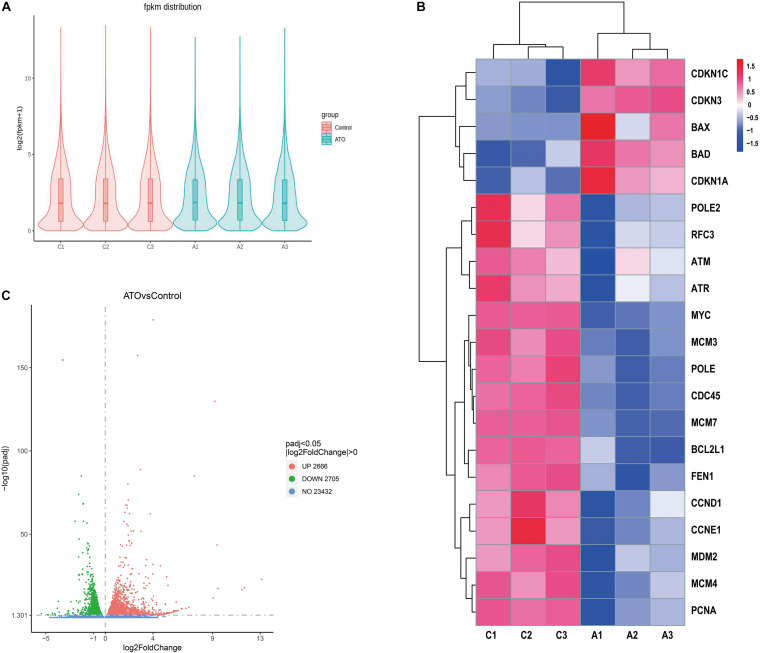
RNA sequencing (RNA-seq) identified the differentially expressed genes (DEGs) in the control group and ATO-treated cells. **(A)** A violin graph displaying the comparisons of gene expression level. Each violin map corresponds to five statistics (the maximum, upper quartile, median, lower quartile, and minimum), and the width of each violin represents the number of genes. FPKM (expected number of fragments per kilobase of transcript sequence per million of base pairs sequenced) was introduced to compare the expression level of genes from different experimental conditions. **(B)** The FPKM hierarchical clustering map (heatmap) of the concerned DEGs. The log10(FPKM + 1) values of the DEGs were normalized to the scale numbers and clustered. Red indicates high expression genes, and blue denotes low expression genes. The color ranges from red to blue indicate the value of log10(FPKM + 1) from large to small. **(C)** The overall distribution of the DEGs is shown using volcanic maps. RNA-seq identified 2,666 up-regulated DEGs and 2,705 down-regulated DEGs. Up-regulated genes [log2(fold change) > 0] are shown in red, and down-regulated genes [log2(fold change) < 0] are shown in green. The DEGs were selected with a padj < 0.05 (padj, adjusted *p*-value).

We compared the gene expression profiles of the control group (No drugs) and the ATO group (2 μM ATO for 24 h). The violin plot shows the distribution state and probability density of gene expression between the control group and the ATO group (Figure 2A). As the volcano map shows, there are in total 5,371 DEGs, of which 2,666 genes are up-regulated and 2,705 genes are down-regulated (Figure 2C). The filter conditions are | log2(fold change)| > 0, adjusted *p* < 0.05. Among these DEGs, we clustered the top up-regulated or down-regulated star genes and important hub genes in apoptosis and cell cycle regulation in ATO treatment in the heatmap (Figure 2B). The heatmap highlighted good repeatability within the same group and distinct differences in the expression levels of the concerned DEGs between the two groups, which contained DNA replication-related genes (MCM3, MCM4, MCM7, and PCNA), apoptosis-related genes (BAX, BAD, and BCL2L1), cell cycle-related genes (CDKN1C, CDKN3, CDKN1A, CCND1, and CCNE1), and PPI key node genes (MCM7, MYC, PCNA, CDC45, MCM3, MCM4, POLE2, POLE, RFC3, and FEN1).

### Functional Analysis of DEGs and PPI Network of DEGs

#### GO Enrichment Analysis of Differential Genes

**FIGURE 3:**
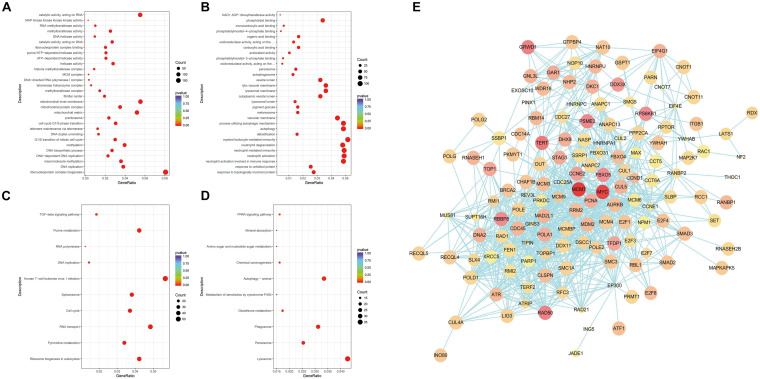
Gene Ontology (GO) and Kyoto Encyclopedia of Genes and Genomes (KEGG) enrichment analysis of differentially expressed genes (DEGs) and protein–protein interaction (PPI) network analysis. **(A)** Scatterplots of down-regulated DEGs with GO enrichment in biological processes, cell components, and molecular functions. **(B)** Scatterplots of up-regulated DEGs with GO enrichment in biological processes, cell components, and molecular functions. **(C)** Scatterplots of down-regulated DEGs with KEGG pathway enrichment. **(D)** Scatterplots of up-regulated DEGs with KEGG pathway enrichment. The degree of GO or KEGG enrichment was measured by the rich factor, *p*-value, and the number of genes enriched on this term. The rich factor refers to the ratio of the number of DEGs (sample) to the number of annotated genes (background) in the term. A term with a *p* < 0.05 is considered an enriched term, and 30 of the most enriched GO terms are shown, whereas 10 of the most enriched KEGG pathways are shown. The size of the dot indicates the number of DEGs. **(E)** PPI network analysis of DEGs. Nodes and edges represent genes and interactions among genes, respectively. The red shade represents the value of log2(fold change), which means the deeper the red is and the larger the log2(fold change) is; the size of the node represents the *p*-value, which means the larger the node is, the smaller the *p*-value is.

Next, the differential genes were divided into three categories through the GO enrichment analysis according to their functions: biological process (BP), cell composition (CC), and molecular function (MF). These differential genes were significantly enriched in 43 BP entries, 22 CC entries, and 19 MF entries (not shown in the figure). Figures 3A,B show the top 10 entries with the most significant differences in GO enrichment (*p* < 0.05, DEGs count ≥ 2). The down-regulated GO terms (Figure 3A) were mainly enriched in processes such as ribonucleic acid–protein complex biogenesis, DNA replication, DNA biosynthesis, methylation, cell cycle G1/S phase transition, and telomere maintenance through telomerase and the up-regulated terms enriched (Figure 3B) were mainly involved in the following processes: response to unfolded proteins and topological error proteins, granulocyte activation and mediated immune response, detoxification process, autophagy and autophagy regulation, and other processes. The GO enrichment results revealed the many terms associated with the killing effect of ATO on KG-1a cells and a series of physiological processes generated by ATO on KG-1a cells. For cell cycle and cell replication, related BPs will be the focus of this article.

#### Pathway Enrichment Analysis of Differential Genes

Compared with the GO enrichment, the KEGG database systematically illustrates the biological functions of gene products in detail. Figures 3C,D show the top 10 entries with the most significant differences in KEGG enrichment (*p* < 0.05, DEGs count ≥ 2). KEGG enrichment results showed that the down-regulated differential genes (Figure 3C) are mainly involved in the following pathways: ribosome production in eukaryotes, purine and pyrimidine metabolism, RNA transport, cell cycle, spliceosome, human T cell leukemia virus type I infection, DNA replication, RNA polymerase, transforming growth factor β pathway, and other related pathways. The up-regulated differential genes (Figure 3D) are mainly involved in the lysosome, peroxisome, phagosome, glutathione metabolism, cytochrome P450 metabolism of foreign substances, autophagy, chemical carcinogenesis function, amino sugar and nucleotide sugar metabolism, mineral absorption, peroxisome proliferator-activated receptor pathway, and other related signal pathways. The KEGG enrichment results suggest that ATO could activate cellular stress responses to promote absorption and metabolism of arsenic, while participating in DNA replication and regulating the expression of key cell cycle genes in KG-1a cells.

#### Analysis of the Interaction Network of DEGs

To clarify the key DEGs involved in cell proliferation, apoptosis, and cell cycle processes, the interactions of DEGs were analyzed by PPI analysis. We found that there are in total 140 nodes and 918 interaction relationships in the PPI network (Figure 3E). In this huge interaction network, we used the CytoHubba plug-in to calculate the degree, closeness, and betweenness of the node genes. Then, we combined the three calculation methods and the relative expression level and *p*-value to select these 10 key node genes (Table 1). These 10 node genes may mainly play an important and complex regulatory role in the PPI network related to cell proliferation, apoptosis, and cell cycle regulation. MYC is located in the most crucial position, followed by MCM7 and PCNA. Thus, MYC, MCM7, and PCNA can be considered as the key genes associated with the ATO-mediated killing effect of KG-1a cells.

### Confirmation of Altered Gene Expression by RT-qPCR

**FIGURE 4:**
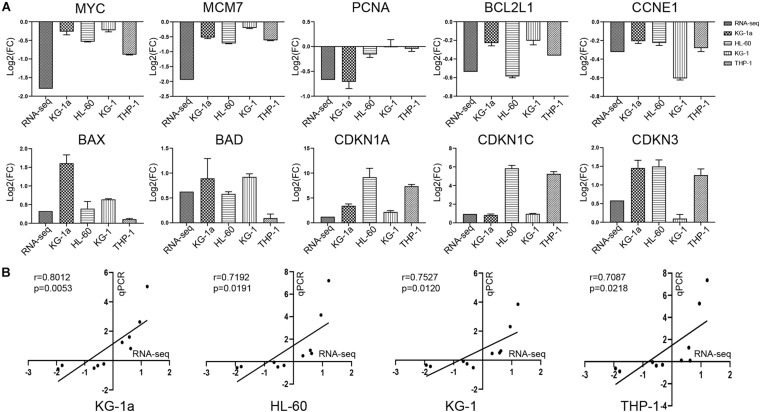
Validation of the RNA sequencing (RNA-seq) with reverse transcription–polymerase chain reaction (RT-PCR). **(A)** Validation of the data with RT-PCR. The 10 genes identified in RNA-seq data were validated in four different AML cells (KG-1a, HL-60, KG-1, and THP-1 cells) by RT-PCR. The data represent the mean ± SD from three experiments. **(B)** Correlation of gene expression ratios between RNA-seq and qRT-PCR in four different AML cells (KG-1a, HL-60, KG-1, and THP-1 cells). Data from both RNA-seq and qRT-PCR were normalized by setting the expression level of the untreated control.

To verify the gene expression identified by RNA-seq, we chose a total of 10 genes in four different AML cells (KG-1a, HL-60, KG-1, and THP-1 cells) for RT-qPCR analysis, and β-actin was selected as a reference gene (Figure 4A). In the comparison of control group versus ATO-treated group, the trend of gene expression was highly consistent with the RNA-seq results. As shown in Figure 4B, the correlation coefficients between the RNA-seq and RT-qPCR data of KG-1a, HL-60, KG-1, and THP-1 cells are 0.8012, 0.7192, 0.7527, and 0.7087, respectively, which means they are highly correlated. These results supported the notion that the RNA-seq data can reflect the changes in transcriptional levels during the ATO-induced apoptosis and cell cycle arrest.

### Confirmation of Altered Gene Expression by Western Blot

**FIGURE 5:**
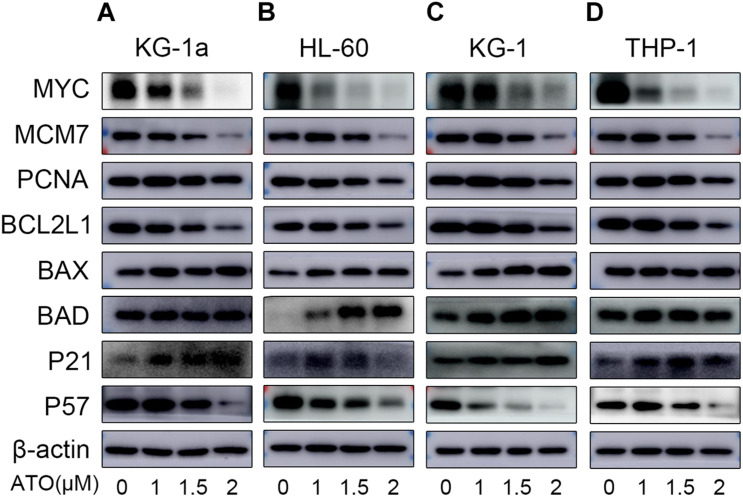
Validation of the RNA sequencing with WB. Validation of the data with WB in four different AML cells (KG-1a, HL-60, KG-1, and THP-1 cells). The representative proteins relative to the DEGs in the DNA replication, cell apoptosis, or cell cycle pathways were examined by Western blotting in KG-1a cells **(A)**, HL-60 cells **(B)**, KG-1 cells **(C)**, and THP-1 cells **(D)**.

As shown in Figures 5A–D, the protein expression of MYC, MCM7, PCNA, and BCL2L1 of four different AML cells was significantly down-regulated, which was in agreement with their mRNA changes in the RT-qPCR. Also, BAX and P21 of four different AML cells were significantly up-regulated consistently with their mRNA changes. However, the protein expression of BAD of KG-1a and THP-1 cells and P57 of four different AML cells was contrary to their mRNA expression (Figures 4A,B), which may be caused by the posttranscriptional regulation, including protein degradation or microRNA regulation.

## Discussion

Arsenic trioxide is a multitarget antitumor drug (Kozono et al., 2018; Alipour et al., 2019; Linder et al., 2019; Xu et al., 2019). This study showed that ATO can inhibit the proliferation of KG-1a cells through inducing apoptosis and cell cycle arresting in the G2/M phase. Through bioinformatics analysis of transcriptome sequencing results, 5,371 DEGs were identified in the ATO group compared to the control group. These DEGs, such as MCM3, MCM4, MCM7, PCNA, and so on, were significantly enriched in the DNA replication pathway. BAX, BAD, BCL2L1, and so on, were related to apoptosis; CCND1, CCNE1, and CDKN1A were related to cell cycle regulation. MYC, MCM7, and PCNA had higher topological scores in the PPI network, and they were the key node genes contributing to the killing effect of ATO on KG-1a cells.

The combined application of ATO and homoharringtonine can significantly increase the apoptosis rate of Kasumi-1 cells (Tan et al., 2019), which is consistent with the results of ATO-promoting KG-1a cell apoptosis in this study (Figures 1B,D). The q-PCR results showed that the proapoptotic genes BAX and BAD were significantly up-regulated, whereas the inhibitory apoptosis gene BCL2L1 was significantly down-regulated (Figure 4A). This result suggests that ATO may promote the apoptosis of KG-1a cells by regulating the transcription level of the BCL family. On the other hand, the cytotoxicity efficacy of ATO to NB4 cells was highest in the G2/M phase and lowest in the G0/G1 phase (Zhou et al., 2018), which indicated that the KG-1a cell cycle arrested in the G2/M phase in this study (Figures 1C,E) may be related to the highest cytotoxicity of KG-1a cells during this period of ATO treatment.

The enrichment results of GO and KEGG suggested that down-regulated differential genes were significantly enriched in the cell cycle and DNA replication process (Figures 3A,C). Because of the large number of differential genes involved in DNA replication, apoptosis, and cell cycle regulation, we analyzed their interaction network and found MYC, PCNA, and MCM7 as the important node genes (Table 1).

MYC is an important proto-oncogene, which is active in many cancers (Dang, 2012). In terms of cell cycle regulation, MYC can promote cell cycle progression by up-regulating cyclin protein expression and down-regulating the expression of inhibitory cyclin protein, resulting in infinite proliferation ability (Garcia-Gutierrez et al., 2019). In this study, we found that the MYC gene and the cyclin genes CCND1 and CCNE1 were significantly down-regulated, and the CDKN1A gene, which encodes the P21 protein, was significantly up-regulated (Figure 4). These results indicated that ATO may attenuate the transcription of CCND1 and CCNE1 and promote the transcription of CDKN1A through down-regulating MYC expression, leading to KG-1a cell cycle arrest in the G2/M phase.

MCM7 is one of the minichromosome maintenance (MCM) protein family members. A previous study showed that the high expression of MCM2, MCM5, and MCM7 might indicate a poor prognosis of cancer (Gou et al., 2018). As a permissive factor for DNA replication, MCM7 can initiate and participate in DNA replication and ensure that DNA replication occurs only once in each cell cycle (Hyrien, 2016). MCM7 gene polymorphism can predict the prognosis of patients with AML (Lee et al., 2017). The researchers additionally reported that ATO can inhibit stem cell proliferation and metabolism of liver cancer cells by targeting the SRF/MCM7 complex (Wang et al., 2019). Another study also demonstrated that silencing MCM7 gene expression by RNA interference technology affects the proliferation and apoptosis of K562 (Tian et al., 2017). These results are consistent with this study (Figure 4), indicating that ATO may inhibit the proliferation of KG-1a cells and promote the apoptosis of KG-1a cells by down-regulating the expression of MCM7.

PCNA is the core component of DNA replication complex and can participate in many important functions such as DNA replication, cell cycle regulation, and DNA damage repair (Moldovan et al., 2007). The study by Wang proved that Mir-363-3p could inhibit the accumulation of endogenous PCNA in lung adenocarcinoma cells, thereby inhibiting lung adenocarcinoma proliferation cells (Zerjatke et al., 2017). Previous studies not only found that fluorescently labeled endogenous PCNA can be used as a marker for cell cycle dynamic analysis (Wang et al., 2017), but also determined that silencing PCNA by siRNA can induce apoptosis of HL-60R cells (Ohayon et al., 2016). These findings provided evidence for our results that ATO may inhibit the proliferation of KG-1a cells and induce apoptosis and cell cycle arrest by inhibiting the expression of PCNA (Figure 4).

In the study of the mechanism of ATO killing hepatoma cells (Wang et al., 2019), the sequencing results of the researchers highly agree with our results (Table 1), which indicated that MCM7 may play an important role in the antitumor effect of ATO. However, the genomic and biochemical data of ATO-induced pancreatic cancer cells have demonstrated that crosstalks between endoplasmic reticulum (ER) stress and autophagy play crucial roles during ATO-induced apoptosis (Xu et al., 2019). In our GO and KEGG enrichment results (Figure 3), autophagy and ER stress response were also activated, indicating that autophagy and ER stress may also play a role in ATO killing AML cells, which may become our next research direction.

In summary, we found that ATO could inhibit the proliferation of KG-1a cells and induce apoptosis and arrest cell cycle. Using transcriptome sequencing technology, we found that the key genes involved in ATO-mediated killing of KG-1a cells were MYC, PCNA, and MCM7. However, the specific mechanism still needs to be further explored. This study will provide new ideas for the clinical treatment of AML with ATO.

## Data Availability Statement

The datasets presented in this study can be found in online repositories. The names of the repository/repositories and accession number(s) can be found below: [CNCB-NGDC (National Genomics Data Center, China National Center for Bioinformation; https://bigd.big.ac.cn/gsa-human/) and accession HRA000642].

## Author Contributions

YL and XN conceived and designed the experiments and wrote the manuscript. YL, YF, and CZ performed the experiments. YL, XN, and XS analyzed the data. FW and XS coordinated the project. All authors contributed to the article and approved the submitted version.

## Conflict of Interest

The authors declare that the research was conducted in the absence of any commercial or financial relationships that could be construed as a potential conflict of interest.

## Publisher’s Note

All claims expressed in this article are solely those of the authors and do not necessarily represent those of their affiliated organizations, or those of the publisher, the editors and the reviewers. Any product that may be evaluated in this article, or claim that may be made by its manufacturer, is not guaranteed or endorsed by the publisher.

## Footnotes

1
https://string-db.org
