# Supplementary material for: Genetic Expression Screening of Arsenic Trioxide-Induced Cytotoxicity in KG-1a Cells Based on Bioinformatics Technology
Source: Front Genet. 2021 Aug 3;12:654826. doi: 10.3389/fgene.2021.654826 (PMC8369888; doi:10.3389/fgene.2021.654826)
Supplement: Supplementary file 1 [file Data_Sheet_1.docx]

Supplementary Material

**Supplementary table 1**. All primers for the RT-qPCR.

| Gene name | Forward primer | Reverse primer |
| --- | --- | --- |
| MCM7 | GAGATTCTCAGCTTCCCCAGG | CTAGCGCGTAGTCCTTCAGT |
| MYC | CGGATTCTCTGCTCTCCTCG | TTCTTGTTCCTCCTCAGAGTCG |
| PCNA | GTAGCAGAGTGGTCGTTGTCT | AGTCTAGCTGGTTTCGGCTT |
| BCL2L1 | CCTTTGCCTAAGGCGGATTTG | TCACTGAGTCTCGTCTCTGGT |
| CCNE1 | TCCTCCAAAGTTGCACCAGT | CGCCACTTAAGGGCCTTCATA |
| BAX | ACCATCATGGGCTGGACATT | GACAGGGACATCAGTCGCTT |
| BAD | TCCCATCGGAAGTTTTGGGT | CAACGGTTAAACCTGGCTCG |
| CDKN1A | CCGAAGTCAGTTCCTTGTGGA | CGCATGGGTTCTGACGGACA |
| CDKN1C | GAGCAGCGTTCGGTTTTGTT | CACCTTGGGACCAGTGTACC |
| CDKN3 | GGACTCCTGACATAGCCAGC | TCTCCCAAGTCCTCCATAGCA |

**Supplementary table 2**. All anti-bodies for the Westernblot.

| Protein name | Antibody name | Company | Molecular weight | Concentration |
| --- | --- | --- | --- | --- |
| MYC | Anti-c-Myc Antibody [Y69] (ab32072) | Abcam, USA | 57 | 1:1000 |
| MCM7 | Anti-MCM7/PRL Antibody [EPR1973Y] (ab134194) | Abcam, USA | 81 | 1:1000 |
| PCNA | PCNA (D3H8P) XP Rabbit mAb #13110 | CST, USA | 36 | 1:1000 |
| BCL2L1 | Bcl-2 (D55G8) Rabbit mAb (Human Specific) #4223T | CST, USA | 26 | 1:1000 |
| BAX | Bax (D2E11) Rabbit mAb #5023 | CST, USA | 20 | 1:1000 |
| BAD | Bad (D24A9) Rabbit mAb #9239 | CST, USA | 23 | 1:1000 |
| P21 | p21 Waf1/Cip1 (12D1) Rabbit mAb | CST, USA | 21 | 1:1000 |
| P57 | p57 Kip2 Antibody #2557 | CST, USA | 57 | 1:1000 |
| β-actin | β-Actin (13E5) Rabbit mAb #4970 | CST, USA | 45 | 1:5000 |
